# Diabetes, Metabolic Syndrome, and Obesity in Relation to Serum Dioxin Concentrations: The Seveso Women’s Health Study

**DOI:** 10.1289/ehp.1206113

**Published:** 2013-05-14

**Authors:** Marcella Warner, Paolo Mocarelli, Paolo Brambilla, Amelia Wesselink, Steven Samuels, Stefano Signorini, Brenda Eskenazi

**Affiliations:** 1 Center for Environmental Research and Children’s Health, School of Public Health, University of California, Berkeley, Berkeley, California, USA; 2 Department of Laboratory Medicine, University of Milano-Bicocca, School of Medicine, Hospital of Desio, Desio-Milano, Italy; 3 School of Public Health, University at Albany, The State University of New York, Albany, New York, USA

**Keywords:** diabetes, dioxin, metabolic syndrome, obesity, Seveso, TCDD, tetrachlorodibenzo-*p-*dioxin

## Abstract

Background: In animal studies, 2,3,7,8-tetrachlorodibenzo-*p*-dioxin (TCDD) alters glucose transport and increases serum lipid levels and blood pressure. Epidemiologic evidence suggests an association between TCDD and metabolic disease.

Objectives: On 10 July 1976, a chemical explosion in Seveso, Italy, resulted in the highest known residential exposure to TCDD. Using data from the Seveso Women’s Health Study (SWHS), a cohort study of the health of the women, we examined the relation of serum TCDD to diabetes, metabolic syndrome, and obesity > 30 years later.

Methods: In 1996, we enrolled 981 women who were newborn to 40 years of age in 1976 and resided in the most contaminated areas. Individual TCDD concentration was measured in archived serum that had been collected soon after the explosion. In 2008, 833 women participated in a follow-up study. Diabetes was classified based on self-report or fasting serum glucose and glycated hemoglobin levels. Metabolic syndrome was defined by International Diabetes Federation criteria. Obesity was defined as body mass index ≥ 30 kg/m^2^.

Results: A 10-fold increase in serum TCDD (log_10_TCDD) was not associated with diabetes (adjusted hazard ratio = 0.76; 95% CI: 0.45, 1.28) or obesity [adjusted odds ratio (OR) = 0.80; 95% CI: 0.58, 1.10]. Log_10_TCDD was associated with metabolic syndrome, but only among women who were ≤ 12 years of age at the time of the explosion (adjusted OR = 2.03; 95% CI: 1.25, 3.29; *p*_interaction_ = 0.01).

Conclusions: We found an increased prevalence of metabolic syndrome associated with TCDD, but only among women who were the youngest at the time of the explosion. Continued follow-up of the SWHS cohort will be informative.

## Introduction

2,3,7,8-Tetrachlorodibenzo-*p*-dioxin (TCDD) is a widespread environmental contaminant and potent endocrine disruptor (Birnbaum and Tuomisto 2000; Zook and Rappe 1994). Endocrine-disrupting compounds are hypothesized to have a direct or indirect role in the pathogenesis of metabolic disorders, including obesity, metabolic syndrome, and diabetes (Casals-Casas and Desvergne 2011; Hectors et al. 2011; Swedenborg et al. 2009). Experimental studies support a link between TCDD exposure and metabolic alterations. TCDD exposure has been associated with wasting syndrome in rodents fed a normal diet (Seefeld et al. 1984), and with accelerated weight gain in mice fed a high-fat diet (Zhu et al. 2008). Effects on glucose homeostasis have also been reported based on *in vivo* and *in vitro* studies, including reduced glucose uptake in adipose tissue, liver, and pancreas; altered glucose tolerance; and impaired insulin secretion (Enan and Matsumura 1993; Enan et al. 1992a, 1992b; Ishida et al. 2005; Kurita et al. 2009). In mice, TCDD exposure has been shown to be associated with increased serum triglycerides, cholesterol, and blood pressure and with earlier onset and greater severity of atherosclerosis (Dalton et al. 2001).

Several epidemiologic studies have examined the relation of exposure to TCDD and other dioxin-like compounds with diabetes, but most were cross-sectional in design and unable to establish temporal relationships between exposures and outcomes. Results from the few longitudinal studies that have been conducted are inconsistent. An increased risk for diabetes has been reported in Vietnam veterans (Kang et al. 2006; Michalek and Pavuk 2008), phenoxyherbicide production workers (Vena et al. 1998), and Yucheng accident cohort members (Wang et al. 2008), but not in Great Lakes sport fish consumers (Turyk et al. 2009), Finnish fishermen and their wives (Turunen et al. 2008), young U.S. adults (Lee et al. 2010), or elderly Swedish adults (Lee et al. 2011a). Higher risks among women than men were reported in the Yucheng cohort (Wang et al. 2008), but few studies have had sufficient power to examine the risk of diabetes in women separately. Recent cross-sectional studies also suggest a positive association of dioxin-like compounds with metabolic syndrome and its individual components (Chang et al. 2010a, 2010b; Lee et al. 2007; Uemura et al. 2009).

On 10 July 1976, a chemical explosion in Seveso, Italy, resulted in the highest known residential exposure to TCDD (Mocarelli et al. 1988). Up to 30 kg of TCDD were deposited over the surrounding 18-km^2^ area (di Domenico et al. 1980), which was divided into exposure zones (A, B, R, non-ABR) based on surface soil TCDD measurements. An ecologic study reported excess mortality from diabetes (1976–2001) among female residents of zone B, the second most contaminated area (*n* = 20 deaths, relative risk = 1.78 [95% confidence interval (CI): 1.14, 2.77)], but not among males (Consonni et al. 2008). However, diabetes was likely underreported as a cause of death, and no individual-level TCDD exposure data were available.

The Seveso Women’s Health Study (SWHS), initiated in 1996, is a historical cohort study of the female population residing around Seveso at the time of the explosion in 1976. The SWHS represents the largest female population with known individual-level TCDD exposure, measured in archived serum that was collected soon after the explosion (Eskenazi et al. 2000). Here, we examine the relation of serum TCDD levels in 1976 with metabolic disorders, including diabetes, metabolic syndrome, and obesity in the SWHS, > 30 years later.

## Methods

*Study population*. Details of the SWHS design have been presented elsewhere (Eskenazi et al. 2000). Briefly, eligible women were newborn to 40 years of age in 1976, resided in the most highly contaminated areas at the time of the explosion, and had adequate stored sera collected soon after the explosion. Enrollment took place from March 1996 to July 1998, and 981 women (80% of those eligible) participated. Between April 2008 and December 2009, we conducted a follow-up of the SWHS cohort: 16 (1.6%) of the women were deceased, 36 (3.7%) could not be located, and 833 (85%) of the original 981 women could be contacted and participated in the follow-up.

For the diabetes analysis, we included the full SWHS cohort (*n* = 981). For the obesity analysis, we included all women who participated in the 2008 follow-up study (*n* = 833). For the metabolic syndrome analysis (*n* = 806), we excluded 27 women who did not undergo a fasting blood draw in 2008.

*Procedure*. The study was approved by the institutional review boards of the participating institutions. Details of the study procedure for the initial study (1996–1998) have been described elsewhere (Eskenazi et al. 2000). Briefly, participation in the initial study included signed informed consent, fasting blood draw, anthropometric measurements, personal interview, medical records, and for most women, a gynecologic examination and ultrasound. Details of the follow-up study (2008–2009) are also described elsewhere (Warner et al. 2011). Participation in the follow-up study included signed informed consent, fasting blood draw, anthropometric and blood pressure measurements, a personal interview including the European Prospective Investigation into Cancer and Nutrition–Italy food frequency questionnaire (Pisani et al. 1997), a memory test, and for a subset, a bone density examination. Additional data were abstracted from medical records. The food frequency questionnaire was analyzed to estimate levels of nutrient components (Pala et al. 2003).

For both the initial and follow-up studies, interviews were conducted in private by trained nurse-interviewers who were unaware of the zone of residence and serum TCDD levels of the participants. During the interviews, a detailed reproductive and medical history was recorded and information on demographic and lifestyle factors was collected. Reproductive information included reproductive diseases, age at menarche, pregnancy history, menopause status, and history of hormone use. Current information on other risk factors included cigarette use, alcohol or caffeine use, physical activity (occupational, recreational, household), and social class factors (education, occupation, income). The medical history included a series of questions about diabetes, medications use, and family history of diabetes.

Anthropometric measurements including height (in centimeters), weight (kilograms), and waist circumference (centimeters) were measured at each study; duplicate measures were made and averaged for analysis. We calculated body mass index (BMI; kilograms per meter squared) and classified women as “overweight” or “obese” if they had a BMI ≥ 25 and < 30 kg/m^2^, or ≥ 30 kg/m^2^, respectively (World Health Organization 1998).

At the 2008 follow-up study, resting blood pressure was measured at three 1-min intervals; and the values were averaged for analysis. Glucose, glycated hemoglobin (HbA1c), triglycerides, and high-density lipoprotein cholesterol (HDL-C) were measured in fasting blood. Metabolic syndrome cases were diagnosed based on the presence of three or more of the following five criteria: *a*) waist circumference ≥ 88 cm; *b*) serum triglycerides ≥ 150 mg/dL; *c*) serum HDL-C < 50 mg/dL; *d*) systolic blood pressure ≥ 130 mmHg or diastolic blood pressure ≥ 85 mmHg or current use of antihypertensive medication; and *e*) serum glucose ≥ 100 mg/dL or report of current use of diabetes medication (Alberti et al. 2009).

Diabetes cases were defined by self-report of diagnosis after 10 July 1976 or by fasting serum glucose and HbA1c levels at the most recent examination. During the interview each woman was asked “Has a doctor ever told you that you had diabetes?” If she answered “yes,” she was asked a series of questions about her age at first diagnosis, if she was first diagnosed during pregnancy, and about her treatment history. If a woman was first diagnosed during pregnancy, but the condition continued after the pregnancy ended, she was considered a diabetes case. If a woman reported she was first diagnosed during pregnancy but had diabetes only during gestation, she was not considered a diabetes case. In addition, women with a current serum glucose ≥ 126 mg/dL or HbA1c ≥ 6.5% were classified as undiagnosed diabetes cases (American Diabetes Association 2011).

*Laboratory analyses*. TCDD was measured in archived sera by high-resolution gas chromatography/high-resolution mass spectrometry methods (Patterson et al. 1987). Details of serum sample selection are presented elsewhere (Eskenazi et al. 2000, 2004). Values are reported on a lipid–weight basis as picograms per gram lipid or parts per trillion (ppt) (Akins et al. 1989). For nondetectable values (*n* = 96), a serum TCDD level of one-half the detection limit was assigned (Hornung and Reed 1990). For the study median serum sample weight of 0.65 g, the median limit of detection was 18.8 ppt, lipid-adjusted.

*Statistical analyses*. Because the serum TCDD distribution was approximately log-normal, TCDD levels were log_10_-transformed. Serum TCDD was analyzed both as a continuous variable (log_10_TCDD) and a four-category variable. The cut point for the lowest category was set at ≤ 20 ppt, because 15–20 ppt was the average TCDD level in serum pools collected from unexposed Italian women in 1976 (Eskenazi et al. 2004). The three remaining categories were defined by calculating tertiles of exposure > 20 ppt, producing groups of ≤ 20, 20.1–47.0, 47.1–135.0, and > 135 ppt.

We used Cox proportional hazards regression models to examine the relation of serum TCDD to occurrence of diabetes. Age was the underlying time variable, with entry defined as the woman’s age on 10 July 1976, and exit defined as her age at diabetes diagnosis (i.e., age at diagnosis for self-reported cases, age at blood draw for undiagnosed cases) or censoring (i.e., age at death, age at 2008 follow-up study for women who participated in 2008, age at 1996 study for women who did not participate in 2008). One woman reported a diabetes diagnosis before the date of the explosion, and therefore was not entered in the Cox models. We used logistic regression to examine the relation of serum TCDD with metabolic syndrome and its individual criteria. We used linear regression to examine the relation of serum TCDD with BMI and polytomous logistic regression to examine the relation of serum TCDD with odds of overweight (BMI ≥ 25 kg/m^2^ and < 30 kg/m^2^) and obesity (BMI ≥ 30 kg/m^2^) as separate outcomes, with underweight/normal (BMI < 25 kg/m^2^) as the reference outcome.

We evaluated a broad range of potential confounding factors identified in the diabetes and metabolic syndrome literature, some of which are listed in Table 1. Covariate information for each woman was based on data collected at last interview. Covariates were included in the final multivariate models if they changed the coefficient for log_10_TCDD by more than 10% or if they were independently associated with the outcome (*p* < 0.10). For all outcomes, we considered effect modification by developmental status at exposure including menarche status at explosion (premenarche vs. postmenarche) and age at explosion (≤ 12 vs. > 12 years). For diabetes and metabolic syndrome, we additionally considered effect modification by obesity (< 30 vs. ≥ 30 kg/m^2^) status. Effect modification was modeled by creating a product term between log_10_TCDD and the effect modifier of interest. Interactions were considered significant if the *p*-value for the product term was < 0.2.

**Table 1:** Select characteristics of the full SWHS cohort (*n* = 981) and the subset (*n* = 833) who also participated in the 2008 follow-up study, SWHS, Italy, 1976–2009.

Characteristic	Full cohort^*a*^ [*n* (%)]	Participants in 2008 study^*b*^ [*n* (%)]
Total	981 (100.0)	833 (84.9)
Characteristics at explosion
Zone of residence
A	167 (17.0)	142 (17.0)
B	814 (83.0)	691 (83.0)
Age (years)
0–10	232 (23.7)	200 (24.0)
11–20	279 (28.4)	252 (30.3)
21–30	241 (24.6)	206 (24.7)
31–40	229 (23.3)	175 (21.0)
Menarche status
Premenarche	284 (29.0)	247 (29.7)
Postmenarche	697 (71.0)	586 (70.3)
Characteristics at last interview
Menopause status
Premenopause	472 (48.1)	394 (47.3)
Postmenopause	509 (51.9)	439 (52.7)
Education^*c*^
≤ Required	651 (66.4)	550 (66.0)
Secondary school	288 (29.4)	249 (29.9)
> Secondary school	42 (4.3)	34 (4.1)
Marital status
Never	76 (7.7)	47 (5.6)
Ever	905 (92.3)	786 (94.4)
Employment status
Employed	481 (49.0)	408 (49.0)
Unemployed	500 (51.0)	425 (51.0)
Cigarette smoking
Never	619 (63.1)	525 (63.0)
Former	194 (19.8)	177 (21.3)
Current	168 (17.1)	131 (15.7)
Alcohol use
Never	618 (63.0)	529 (63.5)
Former	44 (4.5)	32 (3.8)
Current	319 (32.5)	272 (32.7)
BMI categories
Underweight	26 (2.7)	18 (2.2)
Normal	437 (44.5)	352 (42.3)
Overweight	302 (30.8)	263 (31.6)
Obese	216 (22.0)	200 (24.0)
Leisure time physical activity (MET-hr/day)^*d*^
Q1: ≤ 2.3	245 (25.7)	137 (17.0)
Q2: > 2.3–4.7	266 (27.8)	266 (33.0)
Q3: > 4.7–7.3	223 (23.4)	216 (26.8)
Q4: > 7.3	221 (23.1)	188 (23.3)
Family history of diabetes
No	701 (71.5)	564 (67.7)
Yes	280 (28.5)	269 (32.3)
MET, metabolic equivalent of task; Q, quartile. ^***a***^Characteristics at last interview includes data from 2008 for those who participated in the 2008 follow-up study (*n *= 833) and from 1996 for those who did not (*n *= 148). ^***b***^Characteristics at last interview for participants in the 2008 study (*n *= 833) includes data from 2008. ^***c***^≤ Required (≤ 8 years of school), secondary school (9–13 years), > secondary school (> 13 years). ^***d***^Missing data for 26 women (all of whom participated in the 2008 study).		

In sensitivity analyses, we evaluated effect modification by age using alternative age at explosion cut points ranging from 5 to 13 years. We repeated the adjusted models for diabetes excluding three women with possible type 1 diabetes based on reported age at diagnosis and a history of insulin use. We also repeated the final models for diabetes, stratifying on diagnosis status [self-report (*n* = 40) vs. undiagnosed cases (*n* = 14)]. We repeated models of the association between exposure and metabolic syndrome after excluding cases with diabetes (*n* = 40).

For all regression models, standard errors were estimated using the robust Huber–White sandwich estimator. In Cox models, the proportional hazards assumption was tested using scaled Schoenfeld residuals. We conducted tests for linear trend by including categorical TCDD as a continuous term (coded as 0, 1, 2, or 3) in the models. All statistical analyses were performed using Stata Statistical Software, version 11.0 (StataCorp, College Station, TX, USA).

## Results

Demographic characteristics of the 981 women in the SWHS cohort are presented in Table 1. On the date of the explosion, the average age of the full cohort was 20 years, 29% were premenarcheal, and 0.5% were postmenopausal. At last interview, women averaged 50.9 years and about half (52%) were postmenopausal. Most of the women (63%) had never regularly smoked or consumed alcohol, 12.7% reported no leisure-time physical activity [0 metabolic equivalent of task (MET)-hr/day], and 22% were obese. About 29% of women reported a family history of diabetes in a parent or sibling. Characteristics of the 833 women who participated in the 2008 follow-up study were similar to those of the full cohort (see Table 1).

The median lipid-adjusted TCDD concentration measured in blood collected near the time of the explosion in 1976 for the full cohort (*n* = 981) was 55.9 ppt (interquartile range, 28–157). Median serum TCDD levels were higher among women who were youngest at the time of the explosion (165.0 ppt for women 0–10 years of age vs. 45.6 ppt for women 11–40 years of age) or who were still premenarche at the time of explosion (142.5 ppt for premenarche vs. 44.4 ppt for postmenarche), as reported previously (Eskenazi et al. 2004). Median serum TCDD levels were also higher in women who at last interview were never married, more educated, currently employed, or premenopausal, but these likely reflect the higher TCDD levels in younger women (data not shown).

Over the 32-year follow-up period, 54 women (5.5%) in the cohort were diagnosed with diabetes. Of the 54 cases, 40 were identified by self-report of a doctor’s diagnosis and an additional 14 were diagnosed by fasting serum glucose or Hb1Ac levels at the most recent blood draw. The average age at diagnosis of all 54 cases was 54.9 (± 9.4) years. The geometric mean (geometric standard deviation) serum TCDD level for the diabetes cases [47.9 (3.7) ppt] was significantly less than for the noncases [*n* = 926, 71.1 (4.2) ppt] (analysis of variance for log_10_TCDD: *p* = 0.05). As presented in Table 2, the adjusted hazard ratio (HR) for diabetes associated with a 10-fold increase in TCDD (log_10_TCDD) was 0.76 (95% CI: 0.45, 1.28). When TCDD was modeled as a categorical variable, there was some evidence of an inverse dose–response trend (*p* = 0.09). The trend was not monotonic because the hazard of diabetes was nonsignificantly increased in the second TCDD quartile, and it was nonsignificantly decreased in the third and fourth quartiles. We found no evidence of effect modification by obesity status (for nonobese, adjusted HR = 0.68; 95% CI: 0.32, 1.44; for obese, adjusted HR = 0.85; 95% CI: 0.41, 1.78; *p*_interaction_ = 0.68). There were too few cases to examine potential effect modification by menarche status (*n* = 2 cases premenarche) or age at explosion (*n* = 3 cases ≤ 12 years).

**Table 2:** HRs from Cox proportional hazards models for association between 1976 serum TCDD levels and diabetes risk; SWHS, Italy, 1976–2009.

Exposure	No. of cases/total	HR (95% CI)	Adjusted HR^*a*^ (95% CI)
Log_10_TCDD^*b*^	54/980	0.85 (0.50, 1.42)	0.76 (0.45, 1.28)
		*p* = 0.53	*p* = 0.30
TCDD (ppt)			
≤ 20	8/154	1.00	1.00
20.1–47.0	27/275	1.58 (0.71, 3.48)	1.53 (0.70, 3.36)
47.1–135.0	11/278	0.79 (0.32, 1.95)	0.75 (0.32, 1.80)
> 135	8/273	0.84 (0.31, 2.24)	0.66 (0.24, 1.85)
		*p*_trend_ = 0.20	*p*_trend_ = 0.09
^***a***^Adjusted for alcohol consumption, waist circumference, and family history of diabetes. ^***b***^HR for a 10-fold increase in serum TCDD.			

The average BMI of the 833 women who participated in the 2008 follow-up study was 26.7 (± 5.5) kg/m^2^; 263 (32%) women were overweight and 200 (24%) were obese. We excluded one woman with a BMI of 57.5 kg/m^2^ from the linear regression models of BMI in order to prevent an excessive influence on the results. As presented in Table 3, log_10_TCDD was not associated with BMI (adjusted β = –0.31; 95% CI: –0.88, 0.27). When BMI was categorized, compared to normal weight (BMI < 25 kg/m^2^), there was a nonsignificant reduction in odds of overweight [adjusted odds ratio (OR) = 0.85; 95% CI: 0.64, 1.13] and obesity (adjusted OR = 0.80; 95% CI: 0.58, 1.10) per 10-fold increase in serum TCDD. When serum TCDD was categorized, a significant inverse nonmonotonic trend was observed for BMI, overweight, and obesity (*p*_trend_ = 0.05, 0.05, and 0.04, respectively). We found no evidence of effect modification by menarche status at explosion (*p*_interaction_ = 0.94, 0.52, and 0.94) or age at explosion (≤ 12 vs. > 12 years) (*p*_interaction_ = 0.45, 0.65, and 0.38) for BMI, overweight, and obesity, respectively. However, results tended to be farther from the null in women > 12 years compared with women ≤ 12 years at explosion (for example, for BMI, adjusted β = –0.02; 95% CI: –0.84, 0.80 for women ≤ 12 years and adjusted β = –0.46; 95% CI: –1.25, 0.33 for women > 12 years; *p*_interaction_ = 0.45).

**Table 3:** Adjusted β coefficient from linear regression and ORs from polytomous logistic regression models for the association between 1976 serum TCDD levels and BMI, overweight, and obesity; SWHS, Italy, 1976–2009.

Exposure	BMI (kg/m^2^)		Overweight vs. normal weight		Obese vs. normal weight	
	*n* (%)	Adj β^*a*^ (95% CI)	*n* (%)	Adj OR^*a*^ (95% CI)	*n* (%)	Adj OR^*a*^ (95% CI)
Log_10_TCDD^*b*^	832 (100.0)	–0.31 (–0.88, 0.27)	263 (31.6)	0.85 (0.64, 1.13)	200 (24.0)	0.80 (0.58, 1.10)
TCDD (ppt)
≤ 20	132 (15.9)	0.00	48 (36.1)	1.00	42 (31.6)	1.00
20.1–47.0	234 (28.1)	–0.99 (–2.06, 0.09)	81 (34.6)	0.73 (0.43, 1.23)	64 (27.4)	0.57 (0.32, 1.00)
47.1–135.0	232 (27.9)	–1.45 (–2.53, –0.37)	70 (30.2)	0.58 (0.34, 0.97)	50 (21.6)	0.47 (0.26, 0.84)
> 135	234 (28.1)	–1.10 (–2.19, –0.01)	64 (27.4)	0.60 (0.36, 1.01)	44 (18.8)	0.52 (0.29, 0.93)
		*p*_trend_ = 0.05		*p*_trend_ = 0.05		*p*_trend_ = 0.04
^***a***^Adjusted for age at interview, education, and current alcohol consumption. ^***b***^Estimate for a 10-fold increase in serum TCDD.						

In total, 172 (21%) of the 806 women who underwent a fasting blood draw in 2008 met the diagnostic criteria for metabolic syndrome. Of these, about half (52.9%) of the cases met three criteria for diagnosis, 30.8% met four criteria, and 16.3% met all five criteria. The most prevalent criteria were high blood pressure (47.5%) and increased waist circumference (39.1%). As presented in Table 4, the overall adjusted OR for metabolic syndrome with a 10-fold increase in TCDD was close to 1.0 (adjusted OR = 1.05; 95% CI: 0.78, 1.43), but there was evidence of effect modification by age at explosion (*p* = 0.01). There was a significant positive association of metabolic syndrome with log_10_TCDD among women who were ≤ 12 years of age at the time of the explosion (adjusted OR = 2.03; 95% CI: 1.25, 3.30), but no association among women who were > 12 years of age at the time of the explosion (adjusted OR = 0.96; 95% CI: 0.68, 1.35). A similar difference between the older and younger groups was noted for some of the individual criteria. For example, there was effect modification by age at explosion for high blood pressure (for ≤ 12 years of age, adjusted OR = 1.45; 95% CI: 0.95, 2.21; for > 12 years of age, adjusted OR = 0.54; 95% CI: 0.38, 0.77; *p*_interaction_ < 0.01). However, there was little evidence of effect modification by age at explosion for the HDL-C, triglycerides, or glucose indicators.

**Table 4:** Adjusted ORs for the association between 1976 serum TCDD levels and metabolic syndrome and individual criteria,^*a*^ stratified by age at explosion; SWHS, Italy, 1976–2009.

Outcome	All women (*n* = 806)		Age at explosion				
			≤ 12 years (*n *= 268)		> 12 years (*n *= 538)		*p*_interaction_
	*n* (%)	Adj OR^*b*^ (95% CI)	*n*	Adj OR^*b*^ (95% CI)	*n*	Adj OR^*b*^ (95% CI)	
Metabolic syndrome^*c*^	172 (21.3)	1.05 (0.78, 1.43)	16	2.03 (1.25, 3.30)	156	0.96 (0.68, 1.35)	0.01
Waist circumference^*d*^	315 (39.1)	0.98 (0.75, 1.26)	58	1.32 (0.84, 2.06)	257	0.83 (0.61, 1.14)	0.10
Triglycerides^*d*^	101 (12.5)	1.02 (0.71, 1.48)	18	1.31 (0.63, 2.70)	83	0.98 (0.65, 1.49)	0.50
HDL-C^*d*^	202 (25.1)	1.07 (0.81, 1.41)	59	1.35 (0.87, 2.09)	143	0.98 (0.68, 1.40)	0.26
Blood pressure^*d*^	383 (47.5)	0.71 (0.53, 0.94)	44	1.45 (0.95, 2.21)	339	0.54 (0.38, 0.77)	< 0.01
Glucose^*d*^	155 (19.2)	0.94 (0.69, 1.29)	10	0.63 (0.31, 1.27)	145	1.07 (0.76, 1.50)	0.18
^***a***^Individual criteria: waist circumference ≥ 88 cm, serum triglycerides ≥ 150 mg/dL, serum HDL-C < 50 mg/dL, blood ­pressure ≥ 130/85 mmHg or current use of antihypertensive medication, and serum glucose ≥ 100 mg/dL or report of ­current use of diabetes medication. ^***b***^OR for a 10-fold increase in serum TCDD. ^***c***^Adjusted for age at interview, leisure-time physical activity, family history of diabetes, and current use of medication that may increase serum glucose. ^***d***^Adjusted for age at interview.							

We found no evidence of effect modification by menarche status at explosion (*p* = 0.57). In sensitivity analyses, we considered effect modification by other age at explosion cut points, although at younger ages the number of cases was small. For 6–13 years of age, the interaction *p*-value was < 0.20, with an increased risk of metabolic syndrome in the younger group (significant at *p*_interaction_ < 0.05 for ages 11 and 12 years, and at *p*_interaction_ < 0.10 for ages 9, 10, and 13 years). ORs in the younger group ranged from 1.60 to 2.03, and ORs in the older group were ~ 1.00 at all ages. Results were strongest and most significant for the 12-year-age at explosion cut point.

In sensitivity analyses, we repeated the final models for diabetes excluding three possible type 1 diabetes cases (age of onset: 25, 34, and 37 years of age) and stratifying on diagnosis status [self-report (*n* = 40) vs. undiagnosed (*n* = 14)] and the results were similar (data not shown). We repeated the final models for metabolic syndrome excluding diabetes cases (*n* = 40) and the results were similar (data not shown).

## Discussion

To our knowledge, this is the first epidemiologic study to prospectively examine the relation of individual serum TCDD levels and metabolic disorders including diabetes, metabolic syndrome, and obesity in a highly exposed population. In this study of women residing in Seveso, Italy, in 1976, at the time of an explosion that resulted in very high levels of TCDD exposure, we found a significant positive association between metabolic syndrome and serum TCDD levels, but only among women who were youngest at exposure. In contrast, obesity was not associated with TCDD, and this association did not vary with age at exposure. Finally, we found no association of serum TCDD levels with diabetes, but there were too few cases to examine any possible effect modification by age at exposure.

The observed association between TCDD in 1976 and metabolic syndrome in 2008 is consistent with recent cross-sectional studies suggesting a positive association between TCDD and dioxin-like compounds with prevalence of metabolic syndrome (Chang et al. 2010a, 2010b; Uemura et al. 2009). A significant increasing trend in prevalence of metabolic syndrome was reported with increasing quintiles of TCDD and total toxic equivalents (TEQs) [based on polychlorinated dibenzodioxins (PCDDs) and dibenzofurans (PCDFs)] measured in the serum of 1,490 Taiwanese residents living in the vicinity of an abandoned pentachorophenol factory (Chang et al. 2010b). The adjusted ORs for the highest quintiles of TCDD and total TEQ were 2.8 (95% CI: 1.6, 4.9) and 2.3 (95% CI: 1.3, 3.9), respectively. In a population-based study of 1,374 Japanese residents, a significant increasing trend in prevalence of metabolic syndrome was reported with increasing quartiles of total TEQ [based on PCDDs, PCDFs, and dioxin-like polychlorinated biphenyls (PCBs)] (Uemura et al. 2009). The adjusted OR in the highest quartile of total TEQ was 5.3 (95% CI: 2.3, 13.0). Our results are also consistent with the hypothesis that early-life exposure to endocrine disrupting compounds, such as TCDD, increases the risk of metabolic disorders later in life. We observed a relationship between TCDD and metabolic syndrome that was limited to women who were ≤ 12 years of age at exposure. Although median serum TCDD levels were higher in the youngest women, there was a wide range of exposure in both groups (≤ 12 years: 3.4–56,000 ppt, > 12 years: 2.5–6,320 ppt). This range allowed us to estimate the relationship between TCDD and metabolic syndrome in both age groups separately. The prevalence of metabolic syndrome (6%) observed among the youngest women in this study (≤ 12 years at the time of explosion) is almost double the prevalence (~ 3.3%) estimated for similar aged women from a nearby region of Italy (Miccoli et al. 2005).

We did not find a significant association of serum TCDD levels with BMI or obesity. However, our finding of a significant nonmonotonic decreasing trend in BMI associated with higher serum TCDD levels is somewhat consistent with two recent epidemiologic studies of dioxin-like PCBs. In a small prospective nested case–control study, a significant decreasing trend in BMI was associated with dioxin-like PCB-156 (but not other dioxin-like PCBs 105, 118, 157, 167), measured in serum in 90 controls (Lee et al. 2011b). A cross-sectional study of older Swedish residents reported a significant decreasing trend in total body fat mass measured by dual X-ray absorptiometry with increasing quartiles of serum dioxin-like PCBs (126, 169, 156, 157, 189) (Ronn et al. 2011). The results of these studies and the present one demonstrating a negative association between TCDD and BMI are not necessarily contradictory to our observed association of TCDD with waist circumference ≥ 88 cm among women who were ≤ 12 years of age at exposure (adjusted OR = 1.32; 95% CI: 0.84, 2.06) because waist circumference is considered a better marker of central adiposity and “metabolic unfitness” than BMI (Alberti et al. 2009). Furthermore, because central adiposity is a strong risk factor for cardiovascular disease, it is plausible that we would find a relationship with waist circumference and not BMI despite their high correlation (*r* = 0.88).

Our finding of no association of serum TCDD levels with diabetes is not consistent with previous prospective studies that reported a positive association between TCDD exposure and diabetes (Consonni et al. 2008; Kang et al. 2006; Michalek and Pavuk 2008; Vena et al. 1998; Wang et al. 2008) nor with previous reports of stronger associations among women than men (Wang et al. 2008). However, there are a number of possible explanations for the differences in findings. Some studies examined risk of diabetes mortality (Consonni et al. 2008; Vena et al. 1998). There was a wide variation in classification of exposure across studies. Some measured TCDD exposure in serum collected years after the last exposure (Kang et al. 2006; Michalek and Pavuk 2008), others measured contemporary levels of some but not all dioxin-like PCB compounds in serum (Lee et al. 2010, 2011a; Turyk et al. 2009), whereas others reported an ecologic measure such as job category (Kang et al. 2006; Vena et al. 1998; Wang et al. 2008). However, given the association between TCDD and metabolic syndrome observed in the youngest age group, a longer follow-up period is needed in this susceptible group to determine whether these younger women develop diabetes as they age.

There is strong biologic plausibility for the metabolic disruption effects of TCDD. Most effects of TCDD are mediated through the aryl hydrocarbon receptor (AhR) (Hankinson 1995; Okey et al. 1994), which has been implicated as a regulator of energy metabolism. TCDD alters glucose and lipid metabolism in mice (Dalton et al. 2001), modifies expression of genes related to insulin transport and signaling pathways in human adipose tissue (Fujiyoshi et al. 2006), and produces oxidative stress at high concentrations (Kern et al. 2002; Matsumura 2003). In C57BL/6N mice, TCDD has been shown to affect the expression of genes related to hepatic circadian rhythm, cholesterol biosynthesis, fatty acid synthesis, glucose metabolism, and adipocyte differentiation in an AhR-dependent manner (Arsenescu et al. 2008; Sato et al. 2008). It has been hypothesized that TCDD may affect energy metabolism by altering mRNA expression of corticotropin-releasing factor in the central nervous system or by affecting levels of adiposity signals including insulin and leptin (Linden et al. 2010). In addition, several direct and indirect mechanisms, including cross-talk with the estrogen receptor, have been proposed (Casals-Casas and Desvergne 2011; Pascussi et al. 2008). A link between TCDD and diabetes has also been hypothesized through interaction between the AhR and peroxisome proliferator–activated receptor (PPAR) γ–mediated signaling pathways (Remillard and Bunce 2002). The PPARγ is a ligand-activated transcription factor involved in lipid metabolism and homeostasis that has been identified as the molecular target of insulin-sensitizing agents used to treat type 2 diabetes (Smith 2002).

This study has several strengths, including the prospective design and ability to measure multiple outcomes related to metabolic disruption by TCDD. We were able to measure individual serum TCDD concentrations near the time of exposure and preceding the diagnosis of metabolic disease. This study represents the largest female population with known individual-level TCDD exposure. An advantage over the previous ecologic study in the Seveso population is that we were able to measure diabetes occurrence, not mortality, thus eliminating potential biases associated with variations in disease survival. In addition, we were able to collect information on confounding factors during the interview that were not available in other studies.

A limitation of this study is that diabetes cases were diagnosed by self-report; however, we were able to include previously “undiagnosed” cases using standardized criteria for fasting glucose and HbA1c measurements. The small number of diabetes cases in the youngest age group prevented us from examining possible effect modification by age at explosion or menarche status. With aging of the SWHS cohort and continued follow-up, it should become possible to examine evidence of interaction by developmental status at exposure.

## Conclusions

This is the first prospective study to examine the relation of individual serum TCDD levels and metabolic disorders in a highly exposed female population. We estimated a significant positive association between serum TCDD level in 1976 and metabolic syndrome in 2008 among women who were ≤ 12 years of age at the time of exposure. In contrast, obesity was not associated with TCDD levels, regardless of age at exposure. We found no evidence of increased risk for diabetes; however, we were unable to examine effect modification by age at exposure. These results are generally consistent with effects of TCDD that have been noted in animal studies and with greater sensitivity to TCDD during development, continued follow-up as the SWHS cohort ages will be informative.
